# Do Instagram Profiles Accurately Portray Personality? An Investigation Into Idealized Online Self-Presentation

**DOI:** 10.3389/fpsyg.2019.00871

**Published:** 2019-04-24

**Authors:** Elspbeth Harris, Aurore C. Bardey

**Affiliations:** Science Department, Fashion Business School, London College of Fashion, University of the Arts of London, London, United Kingdom

**Keywords:** Instagram, online self-presentation, social network sites, personality, idealization

## Abstract

Instagram users are consistently exposed to the presentation of idealized selves. Although studies have examined online self-presentation in general, little attention has been paid to self-presentation in a visual online setting, such as Instagram. The present investigation examined the extent to which Instagram account holders engage in idealized online self-presentation through a mixed-methods approach. Quantitative results (Study 1) showed a difference between how the observers perceived the Instagram account holders’ personality and the Instagram account holders’ personality self-reports. Qualitative findings (Study 2) revealed four major themes: (1) Selfies as a personality predictor; (2) Faces as a personality predictor; (3) Layout as personality predictor, (4) Misuse of social networks and its consequence for communication. Our results also indicated that the halo effect is integral to the online self-presentational process, suggesting that an aesthetically pleasing Instagram account or account holder will be better received and thought as having particularly positive personality traits.

## Introduction

Anonymity plays a central role in online self-presentation. Past studies have found that in comparison to face-to-face interaction, individuals who engaged in online interaction were abler to express their true selves than offline (Joinson, 2001; Bargh et al., 2002). Bargh et al. (2002) posit that ‘under the protective cloak of anonymity (i.e., the internet) users can express the way they truly feel and think.’ This reasoning insinuates that the lack of a shared social network and frequent contact with those outsides of one’s social circle, as seen on Instagram, allows individuals to reveal negative aspects of their identity online and openly self-disclose. Self-disclosure is any message that an individual communicates to another (Cozby, 1973; Wheeless and Grotz, 1976), or more specifically an individual’s propensity for revealing personal information to others (Derlega and Grzelak, 1979; Archer, 1980; Collins and Miller, 1994).

As strategies of self-presentation tend to focus on repressing aspects of the self or modifying the self to appear more desirable (Berg, 1987; Kelly and McKillop, 1996), the concepts of self-presentation and self-disclosure should be juxtaposed. However, scholars have suggested that users have a proclivity to more openly self-disclose online. Joinson (2001) underlined that individuals with higher private self-awareness and reduced public self-awareness showed higher levels of online spontaneous self-disclosure. It is thought that social networking sites allow for more honest self-disclosure due to their reduced social pressures (Moon, 1998, 2000). Correspondingly, Rheingold (1993) suggests that individuals often do reveal themselves more intimately online due to the anonymity offered by social networks, including the intermediation of screens and virtual profile pseudonyms. Therefore, it is possible that certain social networks, as a relatively anonymous free space, may allow for more open self-expression and honest self-presentation. Similarly, digital identity construction (Nguyen and Alexander, 1996) postulates that online self-presentation may make it possible for individuals to more fully disclose aspects of themselves that are difficult to convey physically.

Moreover, the anonymity of social networking sites may encourage a more honest and intimate self-disclosure (Rubin, 1975). In his study on self-disclosure amongst airplane passengers, Rubin (1975) found that out-of-town participants engaged in lengthier and more intimate self-disclosure compared to residents. His findings suggest that self-disclosure is more likely to happen once one is certain that no further interaction with one’s communication partner will take place. Past researchers have theorized that it is the anticipation of an immediate face-to-face interaction that constrains idealized self-presentation online (Gibbs et al., 2006). This is likely because individuals who are expecting to interact with their communication partner again are more cognizant of the potential social repercussions associated with online misrepresentation. This phenomenon suggests that individuals limit their idealized online self-presentation out of apparent concern with appearing deceitful in subsequent face-to-face interactions (Toma et al., 2008). In this sense, Instagram is seemingly the perfect platform for less honest self-presentation and self-disclosure, as it fosters an environment where interaction with strangers is both commonplace and limited to an online setting.

Similarly, digital identity construction theory (Nguyen and Alexander, 1996) postulates that online self-presentation may make it possible for individuals to more fully disclose aspects of themselves that are difficult to convey physically. Brunskill (2013) suggests that it is social networking sites’ varying levels of anonymity in conjunction with their asynchronous nature that results in a more tactical and generalized self-presentation than found in face-to-face interaction. When users present themselves online, they aim to create idealized online identities (Donath, 1999; Walther et al., 2001; Donn and Sherman, 2002; Bowker and Tuffin, 2003; Yurchisin et al., 2005; Schouten et al., 2007; Manago et al., 2008; Toma et al., 2008; Brunskill, 2013). Indeed, the idealized virtual hypothesis supports the notion that social networks facilitate the presentation of one’s best projection by increasing control over the messages one constructs about the self (Schouten et al., 2007; Manago et al., 2008). Subsequently, individuals are able to strategically present positive, idealized versions of themselves online, instead of in face-to-face interactions (Walther et al., 2001). Brunskill (2013) suggests that it is social networking sites’ varying levels of anonymity in conjunction with their asynchronous nature that results in a more tactical and generalized self-presentation than found in face-to-face interaction. In essence, social networks’ non-contemporaneous nature affords their users’ new-found freedom to experiment with their various self-domains. With more time to deliberate and construct a suitable, desired presentation, online users are granted the freedom to explore playful, fantastical online personas that differ from their real-life identities (Turkle, 1995; Stone, 1996).

Interestingly, recent research has highlighted that personality may influence the ideal self-presentation on social network sites. The Five-Factor Model (FFM), or Big Five model, has been called the most comprehensive and parsimonious model of personality (Costa and McCrae, 1992), and personality psychologists are in agreement that personality can best be encapsulated by the model’s dimensions (Devaraj et al., 2008). The FFM has received significant empirical support for being both broad and efficient (Matthews et al., 2003), and is now acknowledged as the customary measure of personality (McCrae and Costa, 1999; Wehrli, 2008). Although the theoretical and methodological foundations of the model are not entirely without dispute (Block, 2010), the Big Five personality traits predict internet use better than cognitive style does (Davis et al., 1989; McElroy et al., 2007; Devaraj et al., 2008). Among the Big Five personality traits, extraversion (i.e., categorized by gregarious, sociable, and affable behavior) has consistently been found to be the most important personality trait in predicting social network site usage (Correa et al., 2010; Seidman, 2013). Previous findings have indicated that extraverts benefit more from online communication (Amichai-Hamburger et al., 2008). Furthermore, Correa et al. (2010) found a positive correlation between extraversion and social network usage, additionally Ryan and Xenos (2011) report significantly higher levels of self-reported extraversion in social network users compared to non-users. As such, extraverts spend more time on social networks (Wilson et al., 2010), are members of more groups (Ross et al., 2009), present a higher level of activity on Facebook (Michikyan et al., 2014) and have significantly more friends/followers (Amichai-Hamburger and Vinitzky, 2010; Ong et al., 2011) than others do.

It is now considered that those who score highly on neuroticism (i.e., characterized by feelings of distress, anxiety, and introspection) use the internet more heavily than extraverts do (Amichai-Hamburger et al., 2002; Correa et al., 2010). Wehrli (2008) reports a positive relationship between neuroticism and social media usage, stating that those low in emotional stability tend to spend more time online to make themselves as attractive as possible. Moreover, positive correlations have been found between neuroticism, the frequency of social network usage (Ryan and Xenos, 2011), and frequency of online instant messenger usage (Correa et al., 2010). Regarding Facebook, in a past study, those high in neuroticism cited the Facebook ‘wall,’ a virtual space on a personal profile used for interaction, as their favorite component of the social network (Ross et al., 2009). Moreover, in another study, especially neurotic individuals were more likely to self-disclose but post fewer photos on their Facebook profile (Amichai-Hamburger and Vinitzky, 2010), a complete reversal compared to extraverted individuals.

In comparison to extraversion and neuroticism, very limited research has examined the relationship between openness (i.e., a curiosity for artistic pursuits and appreciation for alternative viewpoints) and social networks. Research has shown that people high in openness are more likely to use social media and blogs (Guadagno et al., 2008; Correa et al., 2010). However, unlike extraverts, those who score highly on openness reveal more personal information about themselves on social networks (Moore and McElroy, 2012).

Research is scarce with regard to agreeableness (i.e., a pursuit for social coherence, associated with amiability and cooperation) and social network sites. The studies that have been conducted have produced inconsistent results; some scholars have reported both increasing and decreasing propensities of altruistic behavior online (Kiesler and Kraut, 1999; Swickert et al., 2002), whereas others have suggested cross-situational consistency of social behavior both online and offline (Mikami et al., 2010). In addition, agreeableness has been found to be positively related to the frequency of photo uploading and negatively related to the amount of status updating (Amichai-Hamburger and Vinitzky, 2010; Jain et al., 2016).

Like agreeableness, little research has investigated the relationship between conscientiousness (i.e., typified by self-disciplined and organized conduct) and the social networks. Landers and Lounsbury (2006) report that conscientiousness is negatively related to internet usage. Butt and Phillips (2008) found a similar trend, identifying a significant negative correlation between the trait and time spent on social networks as Seidman (2013) who found that low conscientiousness predicted self-presentational behavior. In addition, Amichai-Hamburger and Vinitzky (2010) indicate that although highly conscientious individuals have more online friends, they tend to post fewer images to social networks overall.

Recent studies have attempted to compare this ideal online self-presentation with the actual self in light of their personality. Research highlighted interested but slightly contradictory results. Counts and Stecher (2009) indicated that people hope to convey personality traits through their online profiles and that users are able to create profiles they feel match their desired self-presentation. However, Gosling et al. (2003) highlighted that social site networks users appear to extend their offline personalities online rather than escaping from or compensating for their offline personality. In particular, Seidman (2013) have demonstrated that neuroticism, agreeableness and extraversion traits are positively associated with the tendency to express one’s actual self. Using self-reports, Back et al. (2010) showed that Facebook profiles reflect the users’ actual personality and a not self-idealized personality.

As a platform, Instagram allows its users to edit and share visual content, such as photos and images. This appeals to young people between the ages of 18 and 29 specifically, who represent over one-third of Instagram users (Duggan and Smith, 2013). Although Instagram shares commonalities with other popular social networking sites, such as Facebook, its design differs in many noteworthy ways. Facebook is an ever-changing hub of social interaction that emulates social dynamics within networks (Tufekci, 2008a). Facebook users characteristically use their own names and interact within a relatively public domain, so that their personal profile constitutes a traceable, online social diary. Conversely, researchers have suggested that Instagram is more focused on self-presentation and self-promotion than on the creation and maintenance of relationships (Sheldon and Bryant, 2016). Indeed, Instagram’s design superficially encourages its users to engage in online, visual self-presentation of their ideal selves (Hu et al., 2014).

As a platform that revolves solely around image editing and sharing, additional pressure is placed on Instagram users to present themselves in an appealing way. As a result, users may engage in a variety of virtually deceptive behaviors to counteract enhanced social insecurities. For example, Instagram users may selectively choose older images in which they look more attractive, or even manipulate the photograph before, while, or after it is taken for their profiles (Hancock and Toma, 2009). Instagram’s digital filters facilitate image alteration as they allow users to enhance their content in various ways. In addition, readily available Photoshop imitation applications allow users to further modify their digital presence by engaging in digital cosmetic surgery. An important motivation for engaging in this visually deceptive behavior is the increased likelihood that the image will be successful online. Past research has suggested that Instagram’s ‘like’-based design serves as a system of positive reinforcement that continually encourages its users to self-present (Dumas et al., 2017).

Moreover, as Instagram accounts are public by default, users are aware that their content is available to a wider audience, one that extends beyond friends and family. This presents a paradox in which self-presentation is not only fostered on Instagram but also reinforced. Principally, there is increased social pressure to self-present as images are more readily available to strangers online, while increased interaction with strangers online also allows for a more strategic, idealized self-presentation due to the lack of social accountability (Walther et al., 2001). Thus, one is more likely to self-present and to continue to do so on Instagram due to frequent, common communication with those outside of one’s immediate social circle. Ostensibly, Instagram differs from other social networks as it not only provides the tools for self-presentation but also reinforces self-presentational behavior under the guise of anonymity. Such a paradigm allows Instagram users to deliberately foster a false presentation through their posts (DePaulo et al., 1996; DePaulo and Kashy, 1998).

Through a mixed-methods approach, the aim of the present study is to elucidate the extent of idealized online self-presentation, particularly whether online profiles accurately portray personality. The objectives of the present study are: (1) to assess online self-presentation using a purely visual social network site, i.e., Instagram; (2) to evaluate potential inconsistencies between self-reported personality (offline) and person’s public self (online presentation); (3) to identify the factors implied in assessing a person’s public self.

## Materials and Methods

To fully address the aim of this research project, presented in the section above, two separate studies were developed.

The first study utilized quantitative survey data to elucidate the extent of online self-presentation on Instagram by assessing potential inconsistencies between self-report (offline) and ideal (online) personality. To this end, two groups of participants were recruited: (Group 1) four female *Instagram account holders*, (Group 2) relative and diverse Instagram observers. Using the same measure, Instagram observer participants (Group 2) were given the task of assessing the Instagram account holder participants (Group 1).

Study 2, a qualitative study, set to further contextualize, corroborate, and expand on the findings from the surveys through a series of semi-structured interviews with Instagram observer participants (Group 2) selected from the study 1. This addition of the qualitative component is a unique aspect of this study: it allowed Instagram observer participants (Group 2) to elaborate on their initial conceptions of the account holders’ (Group 1) online presence and further allowed for the further analysis of the combined quantitative data from the first study. Ethics approval for both studies has been obtained by the Psychology Research Ethics Panel at the London College of Fashion.

### Study 1: Quantitative Study

#### Participants

##### Group 1

Because women spend more time on social networks than men, which they use as social capital to style themselves as attractive (Manago et al., 2008; Tufekci, 2008b; Haferkamp et al., 2012), only female Instagram account holders have been recruited for the present study. Four Instagram account holders were recruited for this study. All Group 1 participants were female and within the age range of 18–29 with a mean age of 23.5 (*SD* = 4.50), based on Instagram’s current age demographics. Instagram account holder participants were the subjects of both aspects of the quantitative and later the qualitative study. Reviews of Instagram accounts were both manifold and thorough to ensure the selection of appropriate participants. All four participants had a public Instagram account and their distinct characteristics. They were selected based on their suitability for the study and recruited via social networking sites. Before volunteering to take part in the study, Instagram account holder participants were made aware that a selection of their Instagram images would be made available to Instagram observer participants as an aid in garnering conclusive perceptions on their personalities. The Group 1 participants gladly granted access to their public Instagram for the purpose of this research and were cognizant of their role within both Studies 1 and 2. Before completing the online survey, all participants were asked to read a participant information sheet and to sign a consent form.

##### Group 2

Instagram observers used a larger group of independent participants with no prior connection to the Instagram account holder participants; hence, they were unaware of both the identity and background of each account holder. Instagram observer participants were recruited through opportunity sampling of friends and family, via an online link posted onto a shared Facebook page. A total of 65 respondents completed the online questionnaire. Group 2 participants’ age ranged from 21 to 64, with a mean age of 35.63 (*SD* = 13.2), with 63.08% of respondents being female and 36.92% being male.

#### Materials

The Ten Item Personality Inventory (TIPI) was used as a measure for personality, although, it was adjusted slightly for the Group 1 Instagram observer participants for the purpose of this study. The TIPI (Gosling et al., 2003) was developed using descriptors from other well-established Big Five instruments. Much like the Five Factor Inventory (FFI), each item of the TIPI consists of two descriptors separated by a comma, using the common stem *‘I see myself as’* or, for the Group 2 Instagram observer participants, *‘I see this person as.’* Both the FFI and the TIPI aim to measure individuals on the Big Five factors: extraversion (categorized by gregarious, sociable, and affable behavior), agreeableness (a pursuit for social coherence, associated with amiability and cooperation), conscientiousness (typified by self-disciplined and organized conduct), neuroticism (characterized by feelings of distress, anxiety, and introspection), and openness to experience (a curiosity for artistic pursuits and appreciation for alternative viewpoints). The TIPI uses 10 items and a 7-point scale instead of a 5-point scale. This scale ranges from 1 (strongly disagree) to 7 (strongly agree), with the mean administration time being 1 min. The scale’s tens items consist of pairs of adjectives, and each item measures one of the dimensions of the Big Five factor model. Two out of the 10 questions from the inventory represent one personality trait. One question represents a low level of scores for the trait, and the other a high level. For example, a low level of scores for extraversion would be ‘reserved, quiet,’ whereas a high level would be ‘extraverted, enthusiastic.’ According to Gosling et al. (2003), to score one must first reverse code the question representing the low-level score, and then take an average of the two questions that make up each scale.

Although it has been argued that this measure is not necessarily as reliable as some other models, past literature has supported the TIPI. In their study, Gosling et al. (2003) report promising results with regard to the TIPI’s convergent and discriminant validity in comparison to the FFI (John et al., 1991). In addition, Ehrhart et al. (2009) also report that the correlations of four out of five TIPI scales with measures of locus of control, self-monitoring, life satisfaction, and the trait of anxiety do not significantly differ from those of the IIP-FFM (average difference 0.052). Using this measure in the present study ensured a higher participant response rate as it reduced the number of potential questions by 136.

Instagram observer participants (Group 2) viewed four screenshot images of the Instagram account holder participants’ (Group 1) screenshot images before completing the edited TIPI. These screenshot images were taken 1 week (on the same day and the same time) before the account holders’ participation to ensure representative examples of the accounts without any interference from either the account holders or the researcher. Instagram’s design currently employs a grid layout; thus, each screenshot image of an account holder’s profile consisted of a panel of 12 images. As Instagram observer participants each viewed four screenshots successively, one for each account holder, each had viewed a total of 48 images by the end of the online survey. Although all screenshots featured the same format with the same number of images, the content was diverse due to each profile’s own individuality. One profile was particularly focused on selfies. Another profile had great artistic influence. Two other profiles featured everyday life pictures, one profile being more focused on building and travels, the other one being more focused on urban life and group pictures. Exhibiting such a varied set of profiles ensured that the study would offer the greatest representation of Instagram accounts possible and highlight a clear distinction between the account holders for Instagram observer participants.

#### Procedure

Instagram account holder participants (Group 1) were asked to complete the TIPI by reporting their own personality. They completed 10 statements consisting of two descriptors separated by a comma, using the common stem ‘I see myself as,’ on a 7-point Likert scale. Before taking the survey, Instagram account holder participants were made aware that they would later be the subject of a separate survey, in which others would be asked to make conclusions on their personality via a screenshot of their current Instagram profile.

Instagram observer participants (Group 2) were asked to make inferences regarding the personalities of the Instagram account holder participants via an online questionnaire. To do so, Instagram observer participants viewed four screenshots of the four Instagram account holder participants’ Instagram profiles in sequence (in the form of a screenshot of the researcher’s Instagram profile). After viewing each profile, Instagram observer participants answered a moderated version of the TIPI. For the purpose of this study, the original measure was moderated so that the initial stem *‘I see myself as,’* as utilized in Part 1, was altered to *‘I see this person as.’* Before completing the questionnaire, the purpose and aim were made aware to Instagram observer participants through a pre-emptive participant information sheet, and consent was obtained through a consent form.

#### Data Analysis

Reverse coding of the TIPI questions allowed for the two items corresponding to each personality trait to be averaged together to form a single score for extraversion, agreeableness, conscientiousness, neuroticism, and openness, respectively, per account holder. As such, account holders each had a self-rating score for each of the five personality traits, and the Instagram observers (Group 2) scored each of the four account holders (Group 1) on each of the five personality traits.

The analysis was realized using the statistical software SPSS. As the data followed a normal curve (Kolmogorov–Smirnov and Shapiro–Wilk tests, *p* > 0.05), parametric tests such as Pearson’s correlations, intraclass correlation, one-sample *t*-tests, and Cohen’s *d* were used. The mean of account holders’ individual self-ratings was treated as a known value; thus, one-sample *t*-tests were done with four different values. Furthermore, Cohen’s *d* effect sizes were also calculated for each comparison to indicate the size of the difference between the mean observers ratings and the actual self-ratings (the observers mean minus the self-rating divided by the observers standard deviation), where 0.20 indicates a small effect, 0.50 a medium effect, and 0.80 a large effect.

### Study 2: Qualitative Study

#### Participants

Six semi-structured interviews were conducted with some Instagram observers from Study 1 (Group 2). These participants were each given the option of a follow-up interview at the end of their online questionnaire, and those who were interested provided their email address and were contacted for recruitment. Based on the one-sample *t*-test results regarding individual differences for both age and gender in observers’ responses (see “Results” section of Study 1), it was decided that an equal number of participants from both spectrums of age distribution and gender would be interviewed. The total sample of six adults was composed of three males and three females in the age range of 23–54, with a mean age of 37.83 (*SD* = 15.5). Before completing the 1:1 interview, all participants were asked to read a participant information sheet and to sign a consent form.

#### Materials

Open questions were used to generate discussion about social networks in general, along with specific factors that contributed to the participants’ original perspectives of the Group 1 account holders. These questions were designed not only to reflect survey items but also to allow for open-ended discussion of the dynamics of online self-presentation perhaps not captured by the survey. For example, participants were asked to elaborate on their original perspectives of account holders and offer a self-guided explanation by answering questions such as *‘Which of the following five personality traits do you believe to be the most dominant for this account holder?’* and ‘*What is it about the images that led you to this conclusion?’* Similarly, they were asked whether other factors contributed to their original perspectives, such as captions or aesthetics, and to reflect on social networks as a whole and their own opinions on online self-presentation.

#### Procedure

Due to the nature of semi-structured interviews, Group 2 participants were given the freedom to discuss the questions in as much or as little detail as they chose. The purpose of this study was explained again and several warm-up questions were used, in which it was emphasized that there were no right or wrong answers and that all opinions were appreciated. The same format and series of open-ended questions were used in each interview. Most interviews lasted approximately 15–25 min and were conducted in person and on different days based on participants’ own availability. All interviews were conducted in private rooms. Comments were recorded and transcribed verbatim. Participants are referred to as Participant 1–6 throughout the study to protect their identity.

#### Data Analysis

Braun and Clarke (2006) thematic analysis was used to interpret interview data. As the patterning across language does not adhere to a particular theory of language, thematic analysis has an inherent flexibility that allows it to provide a rich, detailed, and complex account of data. As an analysis method, it can be applied to a range of theoretical frameworks, in this case, the investigation of online self-presentation. Additionally, in order to enhance the quality and validity of thematic analysis results, a time-based triangulation was carried out. Thus, the space of a week was left between initial coding and subsequent coding to ensure that the emergent themes be valid.

## Results

### Study 1: Quantitative Study

Table 1 overleaf shows the self-ratings of each account holder (Group 1) on each of the five personality traits (extraversion, agreeableness, conscientiousness, neuroticism, openness). Also shown in Table 1 are the mean and standard deviation of the observers’ (Group 2) ratings for each account holder. As can be seen, all account holders (Group 1) engaged in relatively high self-report behavior, ranging between 1.5 and 7.0 on a 7-point Likert scale. However, self-reports for 5 and above were common, especially with regard to the trait of openness.

**Table 1:** Big Five personality self-ratings and observers ratings of four Instagram account holders.

	Extraversion	Agreeableness	Conscientiousness	Neuroticism	Openness
**Account holder 1**					
Self-rating	3.00	6.00	5.00	6.50	7.00
Observers mean	4.61	4.28	4.45	3.83	5.20
Observers *SD*	1.51	1.25	1.07	1.19	1.22
One sample *t*-test	8.70^∗∗∗^	-11.20***	-4.16***	-18.24***	-11.97^∗∗∗^
Cohen’s *d*	1.07	-1.38	-0.51	-2.24	-1.47
**Account holder 2**					
Self-rating	3.50	3.50	6.50	5.00	6.00
Observers mean	5.12	3.06	3.87	4.70	5.91
Observers *SD*	1.37	0.98	1.10	1.02	1.08
One sample *t*-test	9.61^∗∗∗^	-3.63***	-19.48***	-2.41*	-0.69
Cohen’s *d*	1.18	-0.45	-2.40	-0.30*	*n.s.*
**Account holder 3**					
Self-rating	5.00	4.00	4.50	2.00	5.50
Observers mean	5.14	5.64	4.92	2.88	5.13
Observers *SD*	1.10	0.79	0.92	0.96	1.13
One sample *t*-test	1.01	16.94***	3.69***	7.43***	-2.78^∗∗^
Cohen’s *d*	*n.s.*	2.09	0.45	0.91	-0.32
**Account holder 4**					
Self-rating	3.50	5.50	6.50	1.50	6.50
Observers mean	3.61	4.05	5.36	3.62	5.60
Observers *SD*	1.03	1.01	1.18	1.18	1.18
One sample *t*-test	0.90	-11.66***	-9.81***	18.08***	-6.22^∗∗∗^
Cohen’s *d*	*n.s.*	-1.44	-0.97	1.80	-0.77

*One sample *t*-test of observers’ mean compared to account holders’ self -rating ^∗^*p* < 0.5, ^∗∗^*p* < 0.01, ^∗∗∗^*p* < 0.001. Cohen’s *d* for *t*-tests that are not significant are not shown (n.s.).*

A series of one-sample *t*-tests were used to compare the Group 2 observers’ ratings of the personality traits to the self-ratings of each Group 1 account holder. As seen in the one sample *t*-test results in Table 1, across the four different account holders and the five different personality traits, there were significant differences between how the Group 2 observers perceived the Group 1 account holders and the account holders’ self-reports. The Group 2 observers rated account holders 1 and 2 as significantly higher on extraversion. Moreover, three of the four Group 1 account holders were rated significantly lower on agreeableness and conscientiousness by the observers than the account holders themselves, while account holder 3 was rated significantly higher by the observers than by herself. In addition, the Group 2 observers rated account holders 1 and 2 as significantly lower on neuroticism than their self-ratings, while account holders 3 and 4 were rated significantly higher. Three of the four Group 1 account holders were rated significantly lower on openness to experience by the observers than by themselves.

To assess how much the Instagram observer participants (Group 2) agreed *with one another* in their ratings of each personality trait, a series of two-way random effects absolute agreement intraclass correlations were calculated for each of the five personality traits across the four account holders (Group 1). The absolute agreements of the observers were small for these 7-point scales and were only significant for the personality traits of agreeableness, ICC(2,66) = 0.163, *F*(65,195) = 1.425, *p* < 0.05, and openness, ICC(2,66) = 0.466, *F*(65,195) = 1.969, *p* < 0.001, indicating that the participants were somewhat in agreement in how they rated the account holders on these traits. Absolute agreements were not significant for the other personality traits of extraversion, ICC(2,66) = 0.036, *F*(65,195) = 1.050, *p* > 0.05, conscientiousness, ICC(2,66) = 0.153, *F*(65,195) = 1.254, *p* > 0.05, or neuroticism, ICC(2,66) = 0.37, *F*(65,195) = 1.058, *p* > 0.05.

Pearson’s correlations were run to determine whether there were significant relationships between the Group 2 observers’ ratings of the Group 1 account holders and participants’ age. Thus, the focus was solely on Instagram observer participants’ data (Group 2). The analysis showed that the older the Group 2 Instagram observer participants were, the more openness they perceived in Instagram account holder 2 (*r* = 0.249. *p* < 0.05). In addition, the analysis indicated that the older the Group 2 observer participants were, the less neurotic they perceived account holder 4 to be (*r* = -0.297, *p* < 0.05). No other relationships between age and ratings were significant, but the results suggest that older Group 2 participants may have seen more openness in account holder 1 (*r* = 0.208, *p* > 0.05) and more agreeableness in account holder 4 (*r* = 0.206, *p* > 0.05).

The distribution of age did have a positive skew; however, Spearman’s ρ or dividing the Group 2 participants into two groups, one younger than 30 (*n* = 35) and the other 30 years and above (*n* = 31), and running independent samples *t*-tests showed the same significant results noted above [account holder 2 and openness: Spearman’s ρ = 0.250, *p* < 0.05, independent samples *t*-test Levene’s test violated *F*(1,64) = 6.33, *p* < 0.05, equal variances not assumed *t*-test *t*(59) = -2.51, *p* < 0.05; account holder 4 and neuroticism: Spearman’s ρ = -2.57, *p* = 0.037, independent samples *t*-test *t*(64) = 2.20, *p* < 0.05]. This indicates that the results were not just driven by outliers or the oldest few Group 2 individuals.

Independent samples *t*-tests were used to look for any gender differences in how Group 2 participants perceived and rated the accounts, again by focusing solely on Instagram observer participants’ data. The only gender difference was that female (*n* = 43) Group 2 participants perceived account holder 3 as significantly more extraverted than male (*n* = 23) participants did [account holder 3: Levene’s test violated *F*(1,64) = 5.020, *p* < 0.05, equal variances not assumed *t*-test *t*(32) = 2.311, *p* < 0.05].

### Study 2: Qualitative Study

Thematic analysis revealed four major themes: (1) Selfies as a personality predictor; (2) Faces as a personality predictor; (3) Layout as personality predictor, (4) Misuse of social networks and its consequence for communication.

#### Selfies as a Personality Predictor

Use of thematic analysis underlined a clear theme regarding the use of selfies, in which selfie usage conveyed personality aspects to participants. As interviews progressed, it became clear that the types of selfies featured on Instagram play a vital role in establishing specifics of individuality. When asked if the use of selfies influenced participants’ opinions regarding the Group 1 account holders, all Group 2 participants answered yes, with many referencing account holder 2 as a prime example. Although Group 2 participants saw this account holder as a creative person, many also deemed her to be *‘self-centered’* or *‘narcissistic,’* which, when questioned, was found to be partly due to her heavy selfie usage: *‘I think that she may be quite self-obsessed, or very self-obsessed. (Why?) Definitely all the selfies.’*

Our results suggest that the nature of the selfie seems to determine how the personality is received as illustrated by Group 2 participants’ insight regarding account holder 2. Whether or not an account holder is perceived as highly narcissistic truly *‘depends on the selfie.’* It seems that the difference may rest on the distinction between three main types of selfies: selfies taken alone, selfies taken with a partner, and group selfies, or groupies. Selfies taken alone are far more likely to be interpreted by viewers as narcissistic than selfies with others, or groupies. Account holder 2 was seen as more self-obsessed ‘*as the pictures were all of herself and with really similar angles… so I really think she likes taking pictures of herself.’* In contrast, account holder 3, whose account also featured selfies but in this case with others, received an entirely different response: ‘*so that would be more of a fun selfie, I wouldn’t really think they were full of themselves with that type of selfie.’* It seems that the difference of just one additional person in a selfie can radically change how the selfie is received: *‘selfies with friends seem more down to earth, whereas frequent selfies of yourself seem more narcissistic.’*

Furthermore, the frequency of posting selfies also denotes a narcissistic personality to Group 2 viewers, as emphasized in the above quote. It appears that more selfies of the account holder alone emphatically emphasize a more self-centered character: *‘when there are a lot of selfies it seems “very all about me,” quite selfish.’* Indeed, the proclivity to not only take but also to post multiple selfies has been shown to be directly related to narcissistic behavior. However, due to the surplus of available knowledge on the topic and with selfies becoming so overtly synonymous with narcissism, it is possible that today’s culture has become hyperaware of the relationship between the two. This stigma may be especially apparent for the younger generation. Millennial, or those between the ages of 18 and 29, who are Instagram most active users (Duggan and Smith, 2013). With this in mind, millennial may be more critical of narcissism as a personality trait on Instagram due to their exposure to the trait within their own generation, or alternatively, due to the stigma of the relationship between selfies and narcissism, they may be conditioned to recognize it within their own generation. A younger interviewee offered important insight into the dynamic of the generational selfie stereotype: *‘there is very much stigma against taking selfies today, especially from older generations that suggest it’s vanity.’*

#### Faces as a Personality Predictor

The analysis yielded a unanimous set of notions regarding the role of faces in online personality perception. Through discussion, it was established that selfies impact how a person is perceived online via two main channels: the frequency of selfies on an account and whether the selfies include others. However, our results suggest that selfies also allow for a greater understanding of character because they show the person’s face. Indeed, several Group 2 participants explained the monumental difference that the captured face makes in interpreting personality: *‘I think it makes a huge difference if you can see that person …identified the person’s character.’* Account holder 2’s frequent selfie usage garnered a more intense reaction from Group 2 participants than any other account holder did, but it was also a more definitive interpretation. It seems that Group 2 participants struggled less to interpret account holder 2 than others because they could see her face in so many of the images: *‘I saw account holder 2’s face a lot, with or without makeup, and it appeared to me that I could get a sense of her personality because I could see her face.’* In complete contrast, account holder 4 showed her face so little that a couple of participants were unsure of her gender, frequently referring to her as him. Thus, it is clear that participants and other potential viewers were left to speculate as to the account holder’s personality: *‘The one with no selfies* (account holder 4), *you are guessing a personality because there are no visual cues, so you’re essentially guessing the person behind the camera.’* It appears that by lacking in selfies, account holder 4 encouraged participants to create a narrative instead of honestly interpreting her personality. One participant said he *‘instantly made a character’* for account holder 4, in which he painted *an ‘image of a really shy, artsy photographer’* while acknowledging that, ‘*that person could be larger than life and like, loud and boisterous.’* Another thought that by not showing her face, the account holder came across as anxious*: ‘Account holder 4, by not showing any selfies, is more neurotic and more self-conscious, whereas account holders that had more selfies, such as account holder 2, had very low neuroticism I’d say.’* For some, it may be clear that not showing one’s face online means that one has something to hide, a case of introversion: *‘people may show lots of their face if they are extraverted, and introverts may not show their face as much.’* In addition, account holder 1 became an interesting example in this regard, covering her face or turning to the side in several of her images. By partially covering her face, she allowed for more of an insight into her personality than account holder 4 while still retaining a level of mystery, with one participant remarking, *‘I wouldn’t be able to know who the person was though as you can’t really see her face.’*

#### Layout as a Personality Predictor

In addition to the use of selfies and the depiction of faces, the thematic analysis revealed the significance of the Group 1 account’s overall style, filters, and colors account in terms of interpreting personality online. This was particularly the case for account holder 4, whose organized account was described as having *‘very similar tones and colors,’* and as *‘whitewashed,’* and *‘minimalist.’* This color scheme seemed to lend itself to a particular depiction of account holder 4, allowing Group 2 participants to almost entirely, unanimously label her as a conscientious individual. Without a scheme, this would have been a struggle due to the lack of faces in the photographs: *‘the use of color denotes a personality aspect, the use of color is part of someone’s personality.’* In fact, many relied solely on color to make inferences about this account holder: *‘I guess the color scheme, that pastel, whitewashed theme, almost feels sterile in a way and in that sterility there’s a sense of loneliness.’* Each Group 2 participant reiterated the importance of an overall theme and filters to bring together a whole image of someone and inevitably his or her personality: *‘Account holder 4’ if the account had been loads of different bright colors it might have maybe looked less organized … because the color scheme is similar it looks very organized and very peaceful and calming.’* It became clear that the strict color scheme and curation utilized by account holder 4 played an important part in how others saw her: *‘when you have something really well curated, it makes you feel that a person’s personality traits are stronger and consistent. If it had been just different objects it would make it feel more chaotic.’* This suggests that the ability to successfully bring together otherwise disjointed images into one coherent theme also denotes a particular personality.

However, Group 2 participants understood that all four Group 1 accounts *‘have a theme’* but unlike account holder 4 ‘*some of the more colorful accounts …come across as more openness to experience and more extroverted.’* Unlike account holder 4’s subdued account, which was interpreted as *‘organized’* and *‘lonely,’* the bright colors of the other accounts painted entirely different pictures of personality. *‘Account holder 1 is confident and gregarious and kind of likes warm tones, and I think that is supposed to denote light and fun. Account holder 2 is all about very gregarious neon and colors.’* Through the use of light tones, account holder 1 emitted a kind, young aura, whereas account holder 2’s loud neon colors seemed to create an image of a loud, rambunctious individual: *‘Account holder 2, the one with all the selfies, looks very bright and loud, so I’d probably think she was a louder person, the use of filters and color schemes used influence me.’*

Although it is clear that the use of color and filters employed throughout the accounts conveyed certain personality aspects for participants, individual interpretation may also have a part to play. Individual visual preferences may influence how a person sees the Group 1 account holder. Whereas one Group 2 participant may not like the colors and organization of account holder 4, deeming it to be *‘sterile and the account holder lonely,’* another may love these factors and view them as *‘peaceful,’* and the account holder as having *a ‘really good eye and style.’* The ‘what is beautiful is good’ stereotype (Dion et al., 1972 also known as *the halo effect*) regarding the personality attributes associated with physical attractiveness suggests that people think that attractive individuals are more competent in both social and occupational spheres, are better adjusted, and are more socially appealing than less attractive adults. It is possible that, especially in the case of color preferences, a more coherent and attractive account may also influence how one sees the account holder: ‘*the more pleasing the images were to look at in terms of their color scheme, the more I was like “oh, I like this person and their account.”’* As this quote suggests, if the layout and color appeal to the viewer individually, and if the viewer believes the account itself to be beautiful, then the viewer may more positively reflect on the account holder because of this.

#### Misuse of Social Networks and Its Consequence for Communication

Our results indicated that all Group 2 participants utilized social networks in one form or another, the most popular networks being Instagram and Facebook. All had similar motivations for engaging in social network usage, the primary reason is to communicate with friends and family and to engage in the sharing of information, whether it was visual or text-based. A somewhat secondary use was to maintain work connections and networking in that regard. Despite all participants owning an account on similar platforms, however, there were varied perspectives on the social networks themselves. Many thought social networks to be useful in maintaining connections but referenced detrimental side effects. Some had qualms about social networks’ ingrained role in society, stating that *‘sometimes the art of conversation and meeting people and contacting people in person is lost’* and that it has become common in the modern world to be asked *‘what is your social media following, how many followers do you have.’* Others thought social networks to be *‘a little worrying’* or to have *‘the potential for misuse, as far as cyberbullying, fake news and the potential to spread hate.’* One Group 2 participant deliberately avoided Instagram as a social network due to its potential to be used as a self-presentational tool, stating, ‘*that’s why I don’t have Instagram… it can be fake.’* Despite frequenting various social networks, a majority of Group 2 participants seemed to have multiple misgivings regarding the platforms, highlighting various areas for change. One participant hoped to limit how much time people spend online, while many others hoped to control the social network’s effects on mental health and self-esteem in one way or another. Three out of six Group 2 participants sought to limit the potential for cyberbullying, stating that *‘it is a real problem that people think they can hide behind a screen and make comments on other people’* and that *‘cyberbullying can change someone’s outlook on life.’* Participants further commented that *‘cyberbullying should be restricted in some manner,’* but were unsure of how to do so without impleading on censorship, other than to make it *‘more difficult for someone to cyberbully, it should be easier for someone to be banned for harassment.’* One Group 2 participant sought to change how rooted social networks are in self-worth, hoping that *‘people don’t base their self-worth, or how they value themselves, on how well their images do.’* He went on to explain the relationship between social networks and mental health, and the anxiety that goes along with the validation seeking that occurs on these platforms: *‘I hope that people don’t depend on it for too much validation, but I know we totally do.’* Others hoped to change the self-presentational and in turn misrepresentational nature of social networks, stating that *‘social media isn’t such a good representation’* and can be *‘a very deceptive tool.’* In this regard, one participant hoped to change *‘the fact that you can filter everything so it looks different,’* while another hoped to stop the *‘proliferation of false information.’*

All Group 2 participants frequented social networks, with five out of six owning an Instagram account, yet they had strong misgivings regarding the platforms themselves. Each Group 2 interviewee highlighted the addicting nature of social platforms and the current awareness of social network misuse.

Finally, the thematic analysis highlighted a somewhat collective perspective on Instagram. Five out of six Group 2 participants used Instagram with varying degrees of activity. Two out of six Group 2 participants did not post at all on the social network but instead used it to keep up with others’ activities, whereas three others did a post on it, ranging from sporadic to active activity. All said that that they read the captions paired with the imagery when browsing the application, though somewhat subconsciously – *‘I’d say I’d almost read without meaning to’* – and that they focused on ‘*the visual mainly, though.’* The preference for imagery is understandable when considering humans’ biological penchant for imagery; people understand visuals faster because they affect them both cognitively and emotionally. Past studies have found that the human brain deciphers image elements simultaneously, while language is decoded in a linear, sequential manner, taking more time to process. However, consideration of captions may hint at a modern distrust of imagery on Instagram, with one participant suggesting that words *‘give a better sense of what their personality is. If they say something witty, you get more of an impression than just a picture that can be easily doctored.’* Statements such as this highlight the potential for self-presentation on Instagram and distrust in the social network in terms of portrayal of personality and four out of six participants reiterated the sentiment. Many thought that ‘*Instagram portrays what they* (account holders) *want their personality to be seen as,’* and that *‘the best stuff is published, so there is always a false face in that respect, you know it does not show life as it is, so there is always an element of promotion there’* and thus *‘you can draw a lot of conclusions that are not true.’*

The interview process made it apparent that there is an inherent distrust for Instagram as an application, and though for the purpose of this study the Group 2 participants were willing to make inferences on these four Group 1 accounts, the majority did not believe Instagram to be an accurate portrayal of personality due to its potential for self-presentation.

## Discussion

Considering the lack of an existing explanatory framework to account for self-presentational efforts online, this study approached the question of whether Instagram profiles accurately portray personality. The study empirically investigated the proposition that Instagram is indeed utilized for the presentation of idealized online selves, the concept of which appears to be a central determinant in overall social network usage. The aim was assessed using a mixed-methods approach. The statistical analysis alone yielded a diverse set of findings. Across the four different account holders and the five different personality traits, there were indeed significant differences in the Instagram observers’ perceptions. In summary, half of the account holders were viewed as more extraverted, more neurotic, and in the case of account holder 3, both more conscientious and agreeable online than offline. In contrast, three out of four account holders were found to be less agreeable and conscientious, in addition to half appearing less neurotic, online than offline. As can be seen, these differences were not consistent across the four accounts, making it difficult to interpret a consistent pattern in keeping with the hypothesis. However, statistical testing alone suggested no clear indication of ideal self-presentation across accounts. Instead, idealization appeared to occur independently, with different accounts engaging in varying levels of online self-presentation.

Selecting a diverse range of accounts for Study 1, instead of one consistent set of profiles, may have directly influenced this finding. A subsequent qualitative investigation revealed that account holders’ individuality played a crucial role in the interpretation of their personality. Although their views were sometimes incongruent, a majority of participants agreed in their interpretation of account holders, with no account holder being collectively characterized by the same dominant personality trait. In hindsight, utilization of one consistent profile type instead of four unique, contrasting accounts may have yielded a more unanimous interpretation of account holder personality, and thus a clearer pattern to later interpret. However, including an assortment of dissimilar profiles seemed to demonstrate how integral individuality is within the concept of online self-presentation. As elucidated, Instagram users seemingly do not self-present in a collective way; instead, their self-presentations vary across traits and across individuals. Thus, individual differences appear to be of paramount importance in self-presentation online.

Correspondingly, individuality also appears to be at the crux of *interpreting* online self-presentation. It was suggested through both quantitative and qualitative components that individual preference is central to the self-presentational process. Intraclass correlations emphasized a general lack of agreement between the Instagram observers with regard to account holders’ personality, thus indicating a divergence in individual perception. Semi-structured interviews corroborated these findings by suggesting that a lack of agreement may indeed be due to personal preference. Discussion revealed that some participants had a penchant for both particular accounts and their account style, based on their appearance. This is in line with past research that has suggested that appearance plays an important role in the observation of and interaction with others (Siibak, 2009; Wang et al., 2016) and that additionally, attractiveness is a fundamental component of social networks (Siibak, 2009; Ringrose, 2011). In this instance, participants may have been more likely to associate the account holders with more positive traits if they found them, or the composition of their account, to be appealing. These findings seem to indicate that the ‘what is beautiful is good’ stereotype (Dion et al., 1972), also known as *the halo effect*, does play a role in the interpretation of account holders’ online selves. With regard to the aesthetic appeal of the accounts, the color appeared to be a crucial factor. The careful portrayal of color has been shown to specifically influence first impressions (Chang and Lin, 2010). Semi-structured interviews revealed that this extends to an online setting and that particular use of filters or color schemes and layout can convey certain personality aspects of the Instagram account holder to the observers. Thus, regarding the halo effect, the more visually appealing the account is overall, the more likely the account holder is to be perceived as having socially desirable traits. Drawing on past research on the social desirability of FFM traits (Bäckström et al., 2009), the findings seem to suggest that profile observers are more likely to perceive account holders as having socially desirable traits, and specifically extraversion or conscientiousness, if they find the account or account holder to be aesthetically appealing.

In addition, as ‘good looks’ are the most significant factor in determining popularity on social networking sites (Siibak, 2009), and ostensibly in all aesthetic domains, it is likely that a better-looking profile will be more successful in both online and offline circumstances. Feingold (1992) posits that attractiveness increases access to social encounters, which in turn improves social adjustment. As Instagram is frequently utilized for networking purposes, it is possible that the more successful an account is, i.e., the more aesthetically pleasing it is, the more likely the account holder is to be recognized in a professional capacity. In addition, considerable empirical evidence suggests that attractiveness impacts employment decision making, with the result that the more attractive an individual is, the greater the likelihood is that that individual will be hired (Watkins and Johnston, 2000). This is especially so if the account holder is a female, as all four account holders were in this study. This common finding that unattractive females are rated less favorably and as less qualified for positions than attractive females is also known as the ‘beauty is beastly effect’ (Heilman and Saruwatari, 1979). All in all, a more attractive profile holder may receive more occupational interest and have increased chances of securing professional positions, with these chances increasing overall if the account holder is an attractive woman. Overall, in combination with past literature, the findings from both studies emphasize the subjectivity of perception within the realm of self-presentation. As such, the halo effect as a social phenomenon seems to significantly influence the interpretation of users’ online self-presentation.

Furthermore, the present qualitative study highlighted several critical concepts to be particularly explicative. For instance, the semi-structured interviews revealed that images with faces allow for a greater understanding of character, supporting past research that suggests that faces are capable of projecting a variety of integral signals in a wide variety of contexts (Goldman and Sripada, 2005). Participants emphasized the monumental difference that seeing a face made in interpreting personality online, suggesting that if an Instagram user posts images lacking faces, this is not only detrimental with regard to personality perception but also portrays a relatively anxious or neurotic personality to the observers. Thus, photographs of faces ostensibly play an important role in establishing personality online.

In this sense, if a user posts multiple selfies, this allows for a greater understanding of personality due to the ability to see that person’s face. However, despite allowing a clearer determination of character, selfies remain a topic of relative controversy. Participants emphasized the narcissistic nature of selfies, suggesting that if a user engages in selfie posting behavior, he/she is more likely to be perceived as narcissistic by others. This finding is in line with past research that has suggested that narcissistic individuals tend to take more selfies than non-narcissistic individuals do (Halpern et al., 2016). Furthermore, discussion indicated that selfies convey exceedingly confident online characteristics and that this confidence can be interpreted in two ways: as highly extraverted or highly narcissistic. Scholars have emphasized the relationship between extraversion and narcissism in the past, suggesting that the traits are positively correlated with one another (Weiser, 2015; Sorokowski et al., 2016). As narcissists are confident, attention seeking, and exhibitionistic, they are often skilled at navigating new social settings and starting new relationships (Buffardi and Campbell, 2008), especially in an online capacity (Buffardi and Campbell, 2008). As both traits share commonalities regarding online sociability, it is understandable that a confident online self, personified by selfie posting, may be interpreted as both extraverted and narcissistic. Lastly, both the type and frequency of selfies posted to Instagram determine whether or not the user is perceived as narcissistic. Selfies taken alone appear to indicate a narcissistic presentation, whereas selfies with others emphasize a more down-to-earth online persona. Moreover, if an account holder has a proclivity to post selfies and engages in this behavior often, he/she will be believed to be more narcissistic than those who post selfies occasionally. In line with this, past research has suggested that narcissists post more selfies than non-narcissists do (Lee and Sung, 2016). The semi-structured interviews categorically highlighted a sensitivity to selfies. This is most likely due to the current research and resulting stigma surrounding selfies and their propagated relationship with narcissism. Although aware of potential conditioning, most participants still reacted adversely to selfies, suggesting that selfie posting greatly impacts how self-presentation is received. Furthermore, the interview process also identified some inherent presentational differences across accounts, whereby participants were able to recognize account holders’ individual self-presentational efforts.

Firstly, our analysis suggested discrepancies between account holder 1’s offline and online selves, showing the trait of extraversion to be the trait given the highest score by the observers for account holder 1. In contrast, in reality, this account holder bordered on the side of introversion, with the trait of extraversion as her lowest self-reported score. The inconsistencies between account holder one’s self-reports and the observers’ ratings suggest that account holder 1 engaged in idealized self-presentation on the trait of extraversion. The social compensation hypothesis states that individuals who struggle to make social connections in face-to-face interactions will use social networks as a place to enhance their interpersonal lives by creating social relationships online (Schouten et al., 2007). In this instance, account holder 1, a self-reported introvert, may have utilized Instagram as a self-presentational tool to appear extraverted to others, thus enhancing her interpersonal life.

Account holder 2 highlighted a number of interesting elements regarding self-presentation perception. Statistical data indicated that she did present an idealized online self, with the observers perceiving her to be less neurotic and more extraverted than her own self-report suggested. However, our analysis revealed that half of the participants predominantly characterized this account holder as neurotic, and her heavy use of makeup selfies appeared to be a strong influencing factor. Although in some circumstances viewed as an artistic expression, the majority of participants indicated makeup’s self-presentational uses. As past literature has suggested, high neuroticism predicts the taking of more selfies and those (Qiu et al., 2015; Sorokowski et al., 2016) and those low in emotional stability tend to utilize social networks to make themselves appear as attractive as possible. It is likely that participants viewed account holder 2’s excessive makeup usage and selfie behavior as an effort to emphasize attractiveness, and they, therefore, perceived her online self as neurotic. In essence, the qualitative findings indicate that selfies and makeup are both self-presentational tools with the intention of portraying an appealing persona online. The negative connotations associated with appearance-enhancing elements suggest the dislike of online self-presentational behavior.

Notably, a negative reaction to online self-presentation was also emphasized with regard to account holder 3, who was somewhat of an outlier in both quantitative and qualitative data. Participants perceived account holder 3 far more favorably than the other account holders. Our analysis showed that the Instagram observers believed her to be the least neurotic and most agreeable account holder. Corroboration through semi-structured interviews revealed that account holder 3 appeared more authentic online and that her honest online self-presentation was responsible for her more favorable reception. Her photographs with others indicated a sense of both amiability and extraversion, and it was also suggested that her *‘normality’* reflected a more down-to-earth personality and that by engaging in a less idealized self-presentation, she was more relatable to others. It is indeed an interesting indication that the more honest an online self-presentation is, the more positively it is received by others. This is especially so when considering that the intent of online self-presentation is to obtain validation from others (Baumeister, 1982). As the interviews also revealed that participants were cognizant of Instagram’s potential as a self-presentational tool, and thus wary of the platform’s capability to accurately portray personality, it was perhaps their prior awareness or experience with online self-presentation that encouraged a preference for more honest profiles and a negative association with online self-presentation.

Overall, this research contributes to the understanding of online self-presentation on current social networks, thus elucidating the role of personality online particularly using a purely visual online setting such as Instagram. Although the present research offers interesting insights, future work would be needed to further explore self-presentation on Instagram. Indeed, additional research assessing the impact of gender on online self-perception would be interesting. It would be also necessary to further evaluate observers’ consistency in Instagram self-presentation as well as further understand the small agreement between our Group 2 observers. Finally, the authors have chosen to use a short scale, i.e., TIPI, for Study 2. However, conducting a convergent and discriminant validity study with more than one measure of personality would allow to better generalize the results to the Instagram population.

## Author Contributions

EH have carried out the literature review and data collection. EH and AB have designed the research project, analyzed the data, and wrote the manuscript together.

## Conflict of Interest Statement

The authors declare that the research was conducted in the absence of any commercial or financial relationships that could be construed as a potential conflict of interest.
